# Optimization of laccase production from *Marasmiellus palmivorus* LA1 by Taguchi method of Design of experiments

**DOI:** 10.1186/s12896-017-0333-x

**Published:** 2017-02-13

**Authors:** Aiswarya Chenthamarakshan, Nayana Parambayil, Nafeesathul Miziriya, P. S. Soumya, M. S. Kiran Lakshmi, Anala Ramgopal, Anuja Dileep, Padma Nambisan

**Affiliations:** Department of Biotechnology, Cochin University of Science and Technology, Cochin-22, Kerala India

**Keywords:** Laccase, Taguchi DOE, Solid state fermentation, *Marasmiellus palmivorus* LA1, Optimization

## Abstract

**Background:**

Fungal laccase has profound applications in different fields of biotechnology due to its broad specificity and high redox potential. Any successful application of the enzyme requires large scale production. As laccase production is highly dependent on medium components and cultural conditions, optimization of the same is essential for efficient product production.

**Results:**

Production of laccase by fungal strain *Marasmiellus palmivorus* LA1 under solid state fermentation was optimized by the Taguchi design of experiments (DOE) methodology. An orthogonal array (L8) was designed using Qualitek-4 software to study the interactions and relative influence of the seven selected factors by one factor at a time approach. The optimum condition formulated was temperature (28 °C), pH (5), galactose (0.8%*w/v*), cupric sulphate (3 mM), inoculum concentration (number of mycelial agar pieces) (6Nos.) and substrate length (0.05 m). Overall yield increase of 17.6 fold was obtained after optimization. Statistical optimization leads to the elimination of an insignificant medium component ammonium dihydrogen phosphate from the process and contributes to a 1.06 fold increase in enzyme production. A final production of 667.4 ± 13 IU/mL laccase activity paves way for the application of this strain for industrial applications.

**Conclusion:**

Study optimized lignin degrading laccases from *Marasmiellus palmivorus* LA1. This laccases can thus be used for further applications in different scales of production after analyzing the properties of the enzyme. Study also confirmed the use of taguchi method for optimizations of product production.

## Background

Laccases (EC 1.10.3.2; benzenediol: oxygen oxidoreductases) are a major group of ligninolytic enzymes which are present in all the eukaryotic kingdoms described in the five kingdom classification by R.H Whittaker in 1969 [1–5]. Laccases non-specifically catalyse one-electron oxidation of four equivalent substrates concomitant with the four-electron reduction of molecular oxygen to water with the help of a copper containing catalytic apparatus [6, 7]. Physiologically, laccase fulfil diverse roles from plant lignin polymerisation [8] to fungal morphogenesis [9]. Being less substrate specific, energy-saving, and biodegradable, laccases were suitable in the development of highly effective, sustainable, and eco-friendly enterprises [10] in the areas of biofuel production [11], chemical transformation of xenobiotics [12], dye decolourisation [13], as biofuel cells [14], effluent treatment [15], pulp bleaching [16], as biosensors [17] and in general food quality improvement [18, 19]. Any application of laccase requires large scale production of the enzyme preferably in a cost effective manner.

Even though other enzyme production systems prefer submerged fermentation, enzyme production from fungi, especially filamentous fungi is better adapted to Solid state fermentation (SSF) as only SSF offers an adherence surface to filamentous fungi [20]. In SSF, growth and enzyme production occur in inert or natural solid material under near or complete absence of free flowing liquid. SSF have advantages like high volumetric productivity [21], effective utilization of agro industrial wastes as substrates that even mimic the natural living surface of fungi and economy [22] due to its static nature. SSF utilizes materials like orange peel [23], banana waste [24], barley bran [25] and pine apple leaves [26] for useful enzyme production, which otherwise pose solid waste disposal problems. This reutilization is appreciated in the context of sustainable development. However, robust control of parameters (both media composition and cultural conditions) in SSF is difficult particularly on an industrial scale, which explains the failure of adapting successful lab scale production systems to an industrial level in the past [27]. This can be overcome by the thorough optimization of the different factors that influence production. Classical single factor method of optimization is an inadequate choice as it is time consuming [28] and will not yield any outcome regarding the relative influence of any of the involving factors. Statistical methods which also accounts for variations in the production process would be appropriate for optimization. Taguchi method of design of experiment is an approach for optimization of parameters, where the production quality stands intact even in an altered environment [29].

The Taguchi method of Design of Experiments (DOE) was developed by Genechi Taguchi who was involved in modifying the Japanese telephone system [30]. The main aim of this method is to determine the optimal process characteristic that is weakly sensitive to noise factors [31]. The taguchi method operates systematically with fewer trials, thus reducing the time, cost and effort, but offer more quantitative information [32]. The method can work even if the parameters are discrete and qualitative. It functions by reducing the sensitivity of the system [33] through thorough parameter designing. For the purpose, taguchi employs a fractional factorial design in the form of an orthogonal array. This array includes representatives from all possible combinations of selected experimental parameters, which are apt to increase the efficiency and precision and simultaneously reducing any experimental errors [34]. Analysis of individual factor contribution along with their interactive effects eventually leads to the identification of finest factors which was further optimized through Analysis of variance (ANOVA). All these advantages contribute to its greater application in other fields of science especially biotechnology.

A newly isolated strain LA1 from rarely explored species palmivorus, is the laccase producing fungus that is selected in the present study. The strain was found to be utilizing pineapple leaves, an inexpensive, unused agro-residue, as substrate for laccase production. The initial laccase activity expressed by *Marasmiellus palmivorus* LA1 was as good as or even higher than that of the initial activities of some of the other reported fungi [35–38]. The present study applies taguchi method for the optimization of extracellular laccase enzyme production in SSF from the fungi *Marasmiellus palmivorus* LA1. The experimental design comprises of seven different factors that proceeds at two levels with L8 (2^7^) array layout for laccase production. This is the first attempt reported for the optimization of laccase production from any *Marasmiellus palmivorus,* which is generally viewed only in the context of palm pathogens.

## Results and discussion

### Determination of factors

Selection of the appropriate culture factors is the prime key for the success of any optimization process. Here the factors and levels selected were based on the preliminary studies of one factor at a time (OFAT) on laccase production by *Marasmiellus palmivorus* LA1. The selected factors do have an influential role in laccase production as it increases the laccase production from 38.53 to 627.7 IU/mL, which is 16.2 fold during OFAT. Previous studies on different fungal laccases also emphasise the requirement of temperature, pH [39], galactose [40], cupric sulphate [41], inoculum concentration [34], and substrate length [42] for increased laccase production. However, in OFAT only the individual factor contributions are taken into consideration, which may vary during factor interactions in an industrial scale scenario.

### Designing of the matrix experiment

Taguchi method of DOE is an effective statistical plan for studying the optimization of laccase production involving several factors. It is reliable for parameter identification with the added advantage of sparing the cost. Implementation of taguchi through Qualitek-4 (QT4) windows version can be through any of the L-4 arrays with three factors at two levels to L-81 arrays with 40 factors at three levels. In the present study, the L-8 array was designed using Qualitek-4 applied in order to study seven different factors. In this orthogonal array, the control factors and the identified noise factors were varied in such a way to find out a combination where variations in noise no longer affect the overall production [43]. These were called the robust designs and the analysis is called the signal to noise ratio analysis. The signal to noise ratio is linked with quadratic loss function, which in turn assumes significant losses can happen within the specification limit [44]. Such losses within limits are expected and can easily be met. “Bigger is better” quality characteristic provides a single index for the measurable results from multiple criteria.

### Experimentation of the designed matrix

All the 8 trials were carried out under SSF. On experimenting the matrix combination, trial 5, which comprises of temperature - 28 °C, pH - 3, ammonium dihydrogen phosphate -0.05% (w/v), galactose - 0.8% (w/v), cupric sulphate – 3 mM, inoculum concentration (number of mycelial agar pieces) - 4 Nos. and substrate length - 0.05 m yielded maximum production with 659 ± 12 IU/mL, while least production is for the trial 1. Trial 1 includes temperature - 26 °C, pH - 3, ammonium dihydrogen phosphate - 0.03% (w/v), galactose - 0.8% (w/v), cupric sulphate – 1 mM, inoculum concentration (number of mycelial agar pieces) - 4 Nos. and substrate length - 0.03 m (Table 1).

**Table 1:** Experimental trial results of all the eight trials conducted

Trials	Laccase activity (IU/mL ± Standard deviation)
1	455.2 ± 1
2	639.9 ± 24
3	542 ± 17
4	557 ± 1
5	659 ± 12
6	458 ± 68
7	606.4 ± 8
8	505.8 ± 29

### Data analysis

The average of obtained enzyme production, in which each factor is at given level, is described in Table 2. Difference between the average values, L2-L1 indicated the relative influence of the particular factor. Greater the difference in values, better the influence on production. The positive value indicates an increase in production as it moves from level 1 to level 2, while the negative value indicates production decrease during the course from L1 to L2. Thus among the selected factors, cupric sulphate increases the laccase production at level 2, followed by substrate length, inoculum concentration, pH, temperature, ammonium dihydrogen phosphate and galactose. Ammonium dihydrogen phosphate has very less or no effect on laccase production with very similar values at level 1 (54.741) and level 2 (54.746). Galactose on the other hand is showing a slight better production at level 1.

**Table 2:** Main effects of all the selected factors

Sl. No:	Factors	Level 1	Level 2	L2 - L1
1	Temperature (°C)	54.718	54.769	.05
2	pH	54.662	54.825	.163
3	NH_4_H_2_PO_4_ (%)^a^	54.741	54.746	.005
4	Galactose (%)	54.967	54.52	-.447
5	Cupric sulphate (mM)	53.721	55.766	2.045
6	Inoculum concentration	54.626	54.861	.234
7	Substrate length (m)	54.185	55.302	1.116

^a^Percentage against buffer

#### Individual factor interaction

**Fig. 1:**
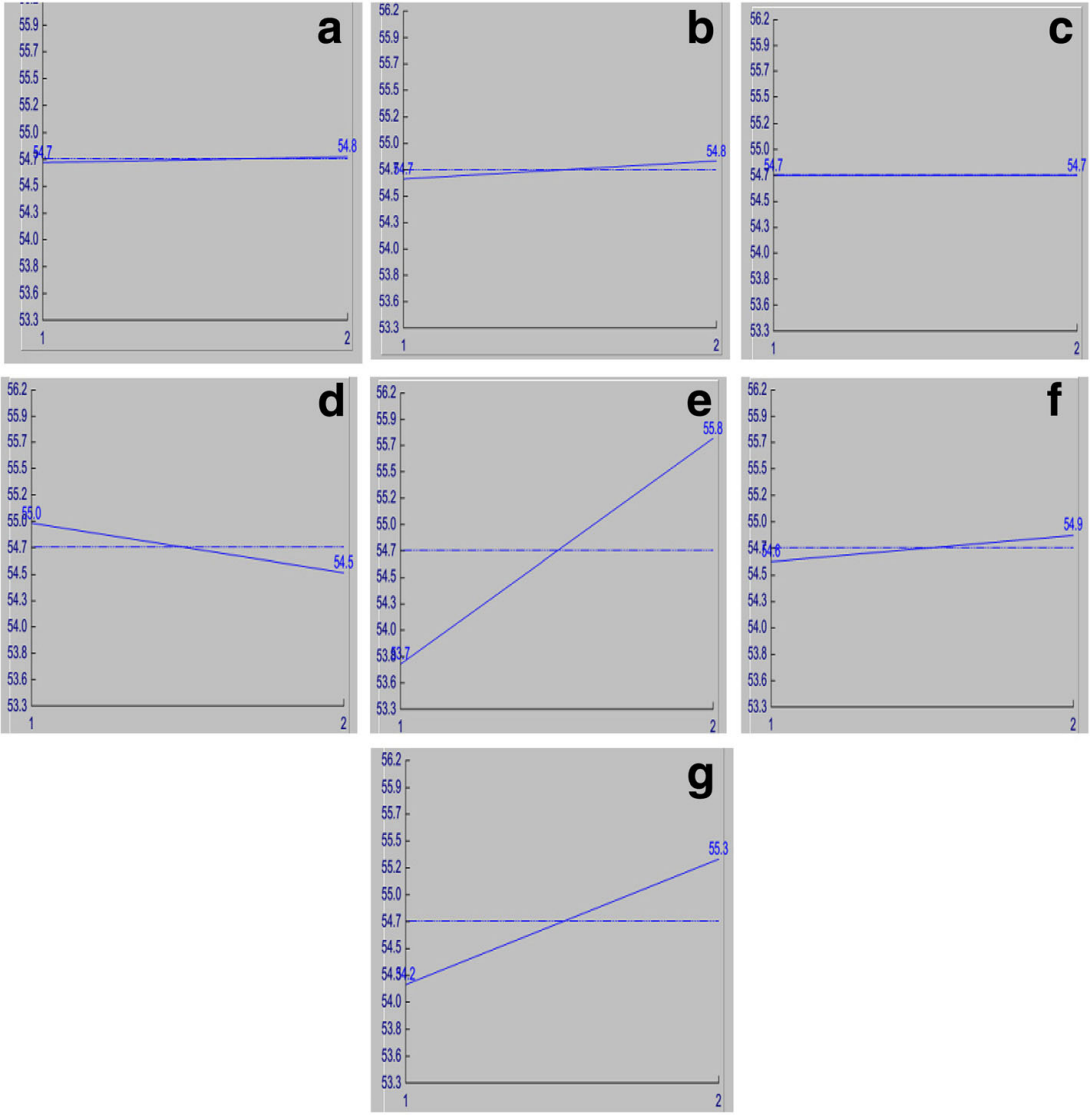
Influence of the selected factors on laccase production by *Marasmiellus palmivorus* LA1. **a** Temperature (°C), **b** pH, **c** Ammonium dihydrogen phosphate (%), **d** Galactose (%w/v), **e** Cupric sulphate (mM), **f** Inoculum concentration (number of mycelial agar pieces), and **g** Substrate length (m). In all the graphs X-axis denotes the different levels (1 and 2) of the concerned factor and Y-axis average effect of the concerned factors

Interaction analysis provides insight into the interaction of a factor with other factors considered during the experiment. The severity index (SI) represents the influence of two individual factors at different levels of interaction. Col. in Table 3 show the position to which interacting factors are allotted. Overall influences of the selected factors on laccase production were depicted graphically (Fig. 1a-g). A perpendicular line represents full (100%) interaction while parallel line means no interaction between the given factors. On analysing the severity index, its noteworthy that ammonium dihydrogen phosphate, the least laccase production influencer interacts maximally with inoculum concentration to give higher severity index (89.72%, Col.5), while the high enzyme production influencing cupric sulphate shows modest interaction with inoculum concentration with low SI (0.19%, Col.3).

**Table 3:** Predicted interactions of the given factors depicted via severity index

Sl. No:	Interacting factor pairs (Order based on SI)	Columns	SI (%)	Col.	Opt.
1	NH_4_H_2_PO_4_ (%) x Inoculum concen.	3 × 6	89.72	5	[1,2]
2	Temperature (°C) x Inoculum concen.	1 × 6	82.66	7	[1,2]
3	Temperature (°C) x Galactose (%)	1 × 4	82.06	5	[2,1]
4	Temperature (°C) x NH_4_H_2_PO_4_ (%)	1 × 3	76.01	2	[2,1]
5	NH_4_H_2_PO_4_ (%) x Galactose (%)	3 × 4	71.42	7	[2,1]
6	pH x Inoculum concen.	2 × 6	65.61	4	[2,2]
7	pH x Substrate length (m)	2 × 7	64.67	5	[1,2]
8	pH x Cupric sulphate (mM)	2 × 5	35.32	7	[1,2]
9	Substrate length (m) x Galactose (%)	2 × 4	34.38	6	[2,1]
10	NH_4_H_2_PO_4_ (%) x Substrate length (m)	3 × 7	28.57	4	[2,2]
11	pH x NH_4_H_2_PO_4_ (%)	2 × 3	23.98	1	[2,1]
12	Galactose (%) x Inoculum concen.	4 × 6	23.92	2	[1,2]
13	Temperature (°C) x Cupric sulphate (mM)	1 × 5	17.93	4	[2,2]
14	Temperature (°C) x Substrate length (m)	1 × 7	17.33	6	[1,2]
15	NH_4_H_2_PO_4_ (%) x Cupric sulphate (mM)	3 × 5	10.27	6	[1,2]
16	Cupric sulphate (mM) x Substrate length (m)	5 × 7	5.15	2	[2,2]
17	Inoculum concen. x Substrate length	6 × 7	3.8	1	[2,2]
18	Temperature (°C) x pH	1 × 2	2.08	3	[2,2]
19	Galactose (%) x Cupric sulphate (mM)	4 × 5	2.06	1	[1,2]
20	Galactose (%) x Substrate length (m)	4 × 7	.28	3	[1,2]
21	Cupric sulphate (mM) x Inoculum concen.	5 × 6	.19	3	[2,2]

Columns: Column locations to which the interacting factors are assigned, SI%: Interaction severity index, Col: Column that should be reserved if this particular interaction is to be studied, Opt.: indicates factor levels desirable for the optimum condition

#### Individual factor contribution and ANOVA

**Fig. 2:**
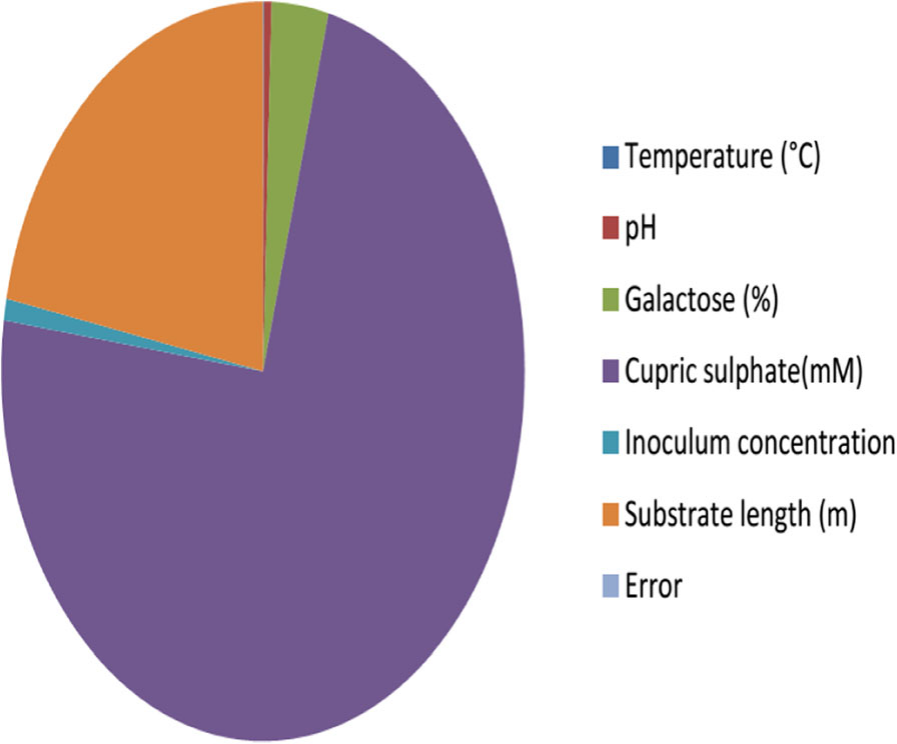
Contribution of each factors on extracellular enzyme production. Cupric sulphate contributes maximum, covering a large area in the figure while ammonium dihydrogen phosphate fails to contribute any

Analysis of variance test was carried out to determine the significance of individual factors on total laccase production (Table 4). Test results showed that cupric sulphate has a significant impact (73.18%) on laccase production followed by substrate length (23.8%). The other factors cumulatively contribute about 4.98% only to laccase production. From the F-ratio of all selected parameters, it was noticed that ammonium dihydrogen orthophosphate has null effect on production thus its effect was pooled. Pooling also helps to avoid saturation of the designed system. All other factors and their interactions considered in the current design were statistically significant at 90% confidence interval indicating that their variability can be explained in terms of significant effects. Contribution of each factor on laccase enzyme production was represented in Fig. 2.

**Table 4:** Analysis of variance (ANOVA)

Sl.No:	Factors	DOF	Sums of squares	Variance	F-Ratio	Pure Sum	Percent
1	Temperature (°C)	1	0.006	0.006	173.585	0.006	0.060
2	pH	1	0.053	0.053	1,325.232	0.053	0.464
3	NH_4_H_2_PO_4_ (%)	1	0		POOLED	0	0
4	Galactose (%)	1	0.401	0.401	10,006.075	0.401	3.510
5	Cupric sulphate (mM)	1	8.366	8.366	208,581.269	8.366	73.181
6	Inoculum concentration	1	0.108	0.108	2,694.782	0.108	0.945
7	Substrate length (m)	1	2.496	2.496	62,237.074	2.496	21.835
	Other/Error	1	0	0			0.005
	Total	7	11.433				100.00%

### Optimum level determination and validation of the optimum

The taguchi method provided optimum culture conditions for each of the influencing factor. The optimum conditions estimated and their contribution are shown in Table 5. Cupric sulphate and substrate length were the major factors affecting laccase production from *Marasmiellus palmivorus* LA1 under solid state fermentation. Signal to noise ratio expected was 56.769 (Table 5), from which the expected production was calculated using the formula, square root (1/Mean Square Deviation (MSD)). MSD represents all the variation around the given target and can be calculated from S/N, where S/N = - Log (MSD). The expected laccase production at optimum conditions was found to be 689.366 IU/mL.

**Table 5:** Optimum culture condition predicted and their contributions on the selected levels

Sl. No:	Factors	Level description	Level	Contribution
1	Temperature (°C)	28	2	0.025
2	pH	5	2	0.081
3	Galactose (%)	0.8	1	0.223
4	Cupric sulphate (mM)	3	2	1.023
5	Inoculum concentration	6	2	0.117
6	Substrate length (m)	0.05	2	0.558
Total contribution from all factors				2.025
Current grand average of performance				54.743
Expected result at optimum condition				56.769

#### Variation reduction plot

**Fig. 3:**
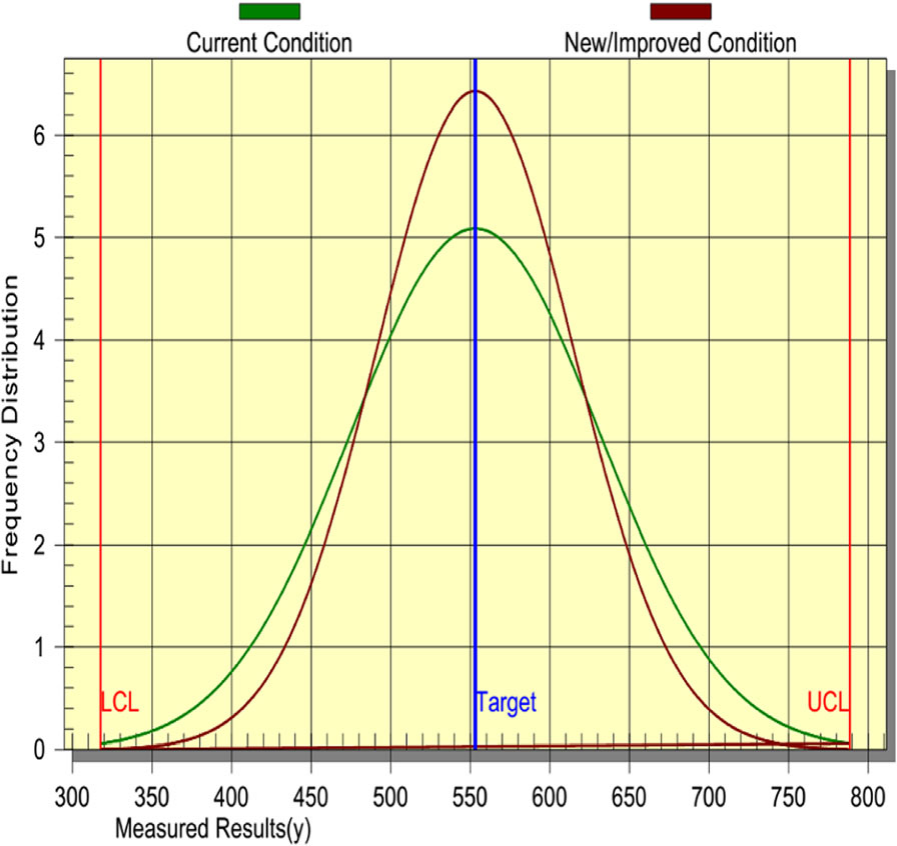
Variation reduction plot based on current and new, improved conditions. Normal performance distribution profiles for laccase activity with higher improved frequency

Variation reduction plot is a graphical representation of the current and improved production status within upper and lower control limits (UCL or LCL) (Fig. 3). Nominal value is 553.062 IU/mL while LCL and UCL being 317.737 and 789.397 IU/mL respectively. Reduced variation is represented by the steep peak in graph. From the graph it’s deducted that the improved condition could cause a savings of 37.3%. This savings owes to the elimination of non necessitated media component.

#### Validation of the optimum

Tests were performed with the optimized factors with the recommended level. This resulted in the production of 667.4 ± 13 IU/mL of enzyme which is comparable to the predicted (689.366 IU/mL). Thus the taguchi method is validated for extracellular laccase production.

Fungal laccase production under solid state fermentation is influenced by various environmental (temperature) and cultural (pH, media components, substrate size, inoculum) conditions [45]. Involvement of many factors leads to optimization to improve the laccase enzyme production. Other than classical approaches, taguchi method of DOE offers a statistical design to create robustness in the process with low lost by considering only the main effects. The method also accounts multiple interaction possibilities between the parameters which is significant in industrial applications. Unlike the past decade, many works were currently relying on taguchi methods for culture parameter optimization [46], process optimization [47, 48], medium optimization [49, 50] and overall yield of enzyme production [51]. In this optimization process the most influencing factors affecting laccase production were found to be cupric sulphate, followed by substrate length and inoculum concentration. Increased production in the presence of copper can be attributed as the defensive response of fungi towards the induced metallic stress [52]. Similar increase in laccase production was observed in *Marasmius quercophilus* in addition of copper [53]. Copper can induce the production of laccase isozymes which leads to increased production [54]. Influence of the pine apple leaf length in production is by offering more surface area and lignin for the basidiomycete, *Marasmiellus palmivorus* LA1 growth and laccase production [55]. Inoculum size does play an important role in establishing the culture in Erlenmeyer flasks, which explains its influential role. The insignificance of ammonium dihydrogen phosphate in the medium was stated through the statistical study, which leads to the elimination of the same from medium, thus reducing the cost without compromising the production quality and quantity. The strain provides a satisfactory yield of laccase (Table 6) leading to the application of the enzyme directly or in immobilized form in industrial settings.

**Table 6:** Comparison of laccase yields of other fungi with the fungus of interest, grown under solid state fermentation

Sl. No:	Organism	Substrate	Enzyme activity (IU/mL)	Reference
1	*Pleurotus ostreatus*	Banana pseudostem	3	[58]
2	*Pleurotus sajor-caju.*	Banana pseudostem	3.6	[58]
3	*Coprinellus disseminatus* SW-1 NTCC 1165	Wheat bran	25.5	[59]
4	*Aspergillus heteromorphus*	Rice straw	6.6	[60]
5	*Aspergillus heteromorphus*	Sugarcane baggase	2.9	[60]
6	*Schizophyllum commune* IBL-06	Banana stalks	345	[61]
7	*Ganoderma lucidum*	Pineapple leaf	472.31 ± 41.2	[26]
8	Coculture of *Pleurotus flabellatus* and *Pleurotus eous*	Coffee pulp	8.8	[62]
9	*Schyzophyllum commune*	Corn stover	130.80	[63]
10	*Pleurotus ostreatus* IBL-04	Wheat straw	517 ± 1.05	[64]
11	*Phanerochaete chrysosporium*	Wheat straw	263.03	[65]
12	*Trametes versicolor* IBL-04	Corn cobs	869.65	[66]
13	*Marasmiellus palmivorus* LA1	Pine apple leaf	667.4 ± 13	Present study

## Conclusion

Optimization of laccase production under solid state fermentation by *Marasmiellus palmivorus* LA1 stain was done via taguchi method of DOE using Qualitek-4. The study aided in the understanding of individual factor contribution and interaction among factors. Elimination of unwanted factors significantly reduce the loss during the process, which otherwise needed to be met. Validation of optimized parameters provides an optimum set of conditions that are insensitive to noise factors which can be used in large scale bioprocess.

## Methods

### Microorganism

For the present study, the culture of fungi *Marasmiellus palmivorus* LA1 isolated from Palakkad district of Kerala, India was used for the production of extra cellular laccases. The strain was grown and then maintained on Potato dextrose agar (PDA) at 4 °C.

### Solid state fermentation for enzyme production

Pineapple leaves of varying length were used as the substrate [26], onto which *Marasmiellus palmivorus* LA1 mycelial agar pieces (0.005 m × 0.005 m sized) were inoculated in 250 mL Erlenmeyer flask. The moisture content was adjusted to 10% with 0.1 M sodium citrate buffer of pH 5. The system was incubated for 5 days under static condition in appropriate temperatures.

### Product extraction

Extracellular enzyme was extracted using 40 mL of 0.1 M sodium citrate buffer of pH 5. After incubation period, the mycelial-free supernatant was collected by gentle shaking followed by centrifugation at 9000 g for 10 min and used for further laccase activity assays.

### Enzyme assay

The laccase assay was performed spectrophotometrically (Shimadzu 1601) at 420 nm using 2,2′-azino-bis (3-ethylbenzothiazoline-6-sulphonic acid) (ABTS) as substrate [56]. One unit (IU/mL) of laccase activity was defined as the amount of enzyme required for the conversion of one micromole of substrate per minute under assay conditions.

### Taguchi method for optimization

In the present study, the taguchi method of optimization moves in five stages: factors determination, matrix designing, experimentation of matrix, data analysis and optimum level validation. All these stages proceed in a stepwise manner to finally yield a valid output.

#### Determination of factors

Using the one factor at a time (OFAT) method for optimization seven different factors that were found to be crucial for laccase enzyme production in *Marasmiellus palmivorus* LA1 were listed out. Then these factors were used for further optimization using taguchi method. The significant influencing factors are temperature, pH, ammonium dihydrogen phosphate (NH_4_H_2_PO_4_), galactose, cupric sulphate, inoculum concentration (number of mycelial agar pieces) and substrate length.

#### Designing of the matrix experiment

QUALITEK-4 software (Nutek Inc., MI, USA) was employed for the purpose [57]. Using an L8 (2^7^) orthogonal array the seven major factors were studied in two levels (Table 7). “Bigger is better” was the quality characteristic preferred in the experimental studies. Signal to noise ratio analysis was used for result analysis.

**Table 7:** Selected culture factors and their assigned levels

Sl.No:	Factors	Level 1	Level 2
1	Temperature (°C)	26	28
2	pH	3	5
3	NH_4_H_2_PO_4_ (%)	0.03	0.05
4	Galactose (%)	0.8	1.2
5	Cupric sulphate (mM)	1	3
6	Inoculum concentration	4	6
7	Substrate length (m)	0.03	0.05

#### Experimentation of the designed matrix

Based on the two levels mentioned (Table 7) 8 different trial sets of solid state fermentation were conducted with *Marasmiellus palmivorus* LA1 (Table 8). All the trials were performed in 250 mL Erlenmeyer flasks having pine apple leaves of length 0.03 or 0.05 m (depending on the assigned levels) wetted with pH 3 or 5 sodium citrate buffer (0.1 M). Ammonium dihydrogen phosphate (0.03% or 0.05% (*w/v*)) and galactose (0.8% or 1.2% (w/v)) were dissolved in the buffer and supplemented. Filter sterilized cupric sulphate was added after autoclaving of the flasks at 121 °C, 1.03 bar pressure for 20 min. Variation in the inoculum concentration was created by using different number of agar pieces (4 or 6). 26 °C or 28 °C temperature was maintained throughout the period of 5-day incubation. Enzyme extraction was performed as previously described. All the trials were performed in triplicates.

**Table 8:** L-8 orthogonal array design

Trials	Columns						
	1	2	3	4	5	6	7
Trial 1	1	1	1	1	1	1	1
Trial 2	1	1	1	2	2	2	2
Trial 3	1	2	2	1	1	2	2
Trial 4	1	2	2	2	2	1	1
Trial 5	2	1	2	1	2	1	2
Trial 6	2	1	2	2	1	2	1
Trial 7	2	2	1	1	2	2	1
Trial 8	2	2	1	2	1	1	2

#### Data analysis

Analysis of the obtained results was done using Qualitek-4 software to infer the interactions between different factors and to give idea about the influence of each individual factor on enzyme production.

#### Optimum level determination and validation of the optimum

By analysing the interactions, the software predicts an optimum condition for maximum enzyme production. The software recommended optimum condition was validated by conducting solid state fermentation and assay testing in triplicates under the optimum condition.

## Abbreviations

ABTS: 2, 2′-azino-bis (3-ethylbenzothiazoline-6-sulphonic acid)
ANOVA: Analysis of variance
DOE: Design of experiments
LSL: Lower specification limit
MSD: Mean square deviation
OFAT: One factor at a time
PDA: Potato dextrose agar
SI: Severity index
SSF: Solid state fermentation
USL: Upper specification limit
